# 1,1′,2,2′-Tetra­methyl-3,3′-(*p*-phenyl­enedimethyl­ene)diimidazol-1-ium bis­(tetra­fluoridoborate)

**DOI:** 10.1107/S1600536809026312

**Published:** 2009-07-11

**Authors:** Subramaniam Puvaneswary, Yatimah Alias, Seik Weng Ng

**Affiliations:** a Department of Chemistry, University of Malaya, 50603 Kuala Lumpur, Malaysia

## Abstract

The title imidazolium-based ionic-liquid salt, C_18_H_24_N_4_^2+^·2BF_4_^−^, has the cation lying about a center of inversion. The five-membered imidazole ring is approximately perpendicular to the six-membered phenyl­ene ring [dihedral angle = 86.9 (1)°]. The tetra­fluoro­borate anion is disordered over two sites in a 0.722 (3):0.278 (3) ratio.

## Related literature

For background to imidazolium-based ionic liquid salts, see: Ganesan *et al.* (2008 ▶). 
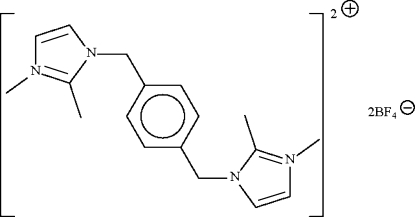


## Experimental

### 

#### Crystal data

C_18_H_24_N_4_^2+^·2BF_4_^−^
*M* *_r_* = 470.03
Monoclinic, 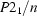

*a* = 8.9095 (2) Å
*b* = 10.2254 (2) Å
*c* = 11.7113 (3) Å
β = 93.024 (1)°
*V* = 1065.45 (4) Å^3^
*Z* = 2
Mo *K*α radiation
μ = 0.14 mm^−1^
*T* = 140 K
0.40 × 0.35 × 0.05 mm

#### Data collection

Bruker SMART APEX diffractometer
Absorption correction: multi-scan (*SADABS*; Sheldrick, 1996 ▶) *T*_min_ = 0.948, *T*_max_ = 0.993
7256 measured reflections
2418 independent reflections
2063 reflections with *I* > 2σ(*I*)
*R*_int_ = 0.017

#### Refinement

*R*[*F* ^2^ > 2σ(*F* ^2^)] = 0.047
*wR*(*F* ^2^) = 0.140
*S* = 1.03
2418 reflections
193 parameters
124 restraints
H-atom parameters constrained
Δρ_max_ = 0.42 e Å^−3^
Δρ_min_ = −0.22 e Å^−3^

### 

Data collection: *APEX2* (Bruker, 2008 ▶); cell refinement: *SAINT* (Bruker, 2008 ▶); data reduction: *SAINT*; program(s) used to solve structure: *SHELXS97* (Sheldrick, 2008 ▶); program(s) used to refine structure: *SHELXL97* (Sheldrick, 2008 ▶); molecular graphics: *X-SEED* (Barbour, 2001 ▶); software used to prepare material for publication: *publCIF* (Westrip, 2009 ▶).

## Supplementary Material

Crystal structure: contains datablocks global, I. DOI: 10.1107/S1600536809026312/tk2495sup1.cif

Structure factors: contains datablocks I. DOI: 10.1107/S1600536809026312/tk2495Isup2.hkl

Additional supplementary materials:  crystallographic information; 3D view; checkCIF report

## Figures and Tables

**Fig. 1.**
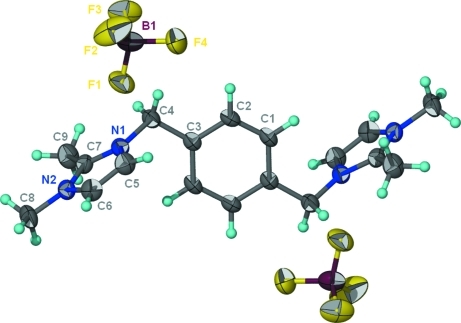
Thermal ellipsoid plot (Barbour, 2001) of [C18H24N4]2+ 2[BF4]- at the 70% probability level; hydrogen atoms are drawn as spheres of arbitrary radius. The [BF4]- anion is disordered; the minor component of the disorder is not shown. The non-H atoms comprising the asymmetric unit are labelled and the unlabelled atoms are related by 1-x, 1-y, -z.

## supplementary crystallographic information

### Experimental

α,α-Dibromo-*p*-xylene (0.78 g, 3 mmol) and 1,2-dimethylimidazole (0.58 g, 6 mmol) were refluxed in DMF (50 ml) for 3 h. The product that separated from solution was collected and washed with ether. Crystals were grown from its solution in water.

The bromide salt (0.46 g, 1 mmol) and sodium tetrafluoroborate (0.11 g, 1 mol) were stirred in water (100 ml) for 24 h. The product that separated from solution was collected and washed with ethanol. Crystals were grown from its solution in DMF.

### Refinement

The [BF_4_]^-^ anion is disordered in both the boron and fluorine atoms. The B–F distances were restrained to within 0.01 Å as were the F···F distances. The disorder refined to a 0.722 (3):0.278 (3) ratio. The anisotropic displacement parameters of the minor component atoms were restrained to be nearly isotropic. The F–B–F angles, although not ideal, are regarded as being satisfactory.

Carbon-bound H-atoms were placed in calculated positions (C—H 0.95 to 0.99 Å) and were included in the refinement in the riding model approximation, with *U*(H) set to 1.2 to 1.5*U*_eq_(C).

### Crystal data

**Table:**

C_18_H_24_N_4_^2+^·2BF_4_^−^	*F*(000) = 484
*M**_r_* = 470.03	*D*_x_ = 1.465 Mg m^−^^3^
Monoclinic, *P*2_1_/*n*	Mo *K*α radiation, λ = 0.71073 Å
Hall symbol: -P 2yn	Cell parameters from 3223 reflections
*a* = 8.9095 (2) Å	θ = 2.6–28.2°
*b* = 10.2254 (2) Å	µ = 0.14 mm^−^^1^
*c* = 11.7113 (3) Å	*T* = 140 K
β = 93.024 (1)°	Irregular block, colorless
*V* = 1065.45 (4) Å^3^	0.40 × 0.35 × 0.05 mm
*Z* = 2	

### Data collection

**Table:**

Bruker SMART APEX diffractometer	2418 independent reflections
Radiation source: fine-focus sealed tube	2063 reflections with *I* > 2σ(*I*)
graphite	*R*_int_ = 0.017
ω scans	θ_max_ = 27.5°, θ_min_ = 2.7°
Absorption correction: multi-scan (*SADABS*; Sheldrick, 1996)	*h* = −11→11
*T*_min_ = 0.948, *T*_max_ = 0.993	*k* = −12→13
7256 measured reflections	*l* = −15→15

### Refinement

**Table:**

Refinement on *F*^2^	Primary atom site location: structure-invariant direct methods
Least-squares matrix: full	Secondary atom site location: difference Fourier map
*R*[*F*^2^ > 2σ(*F*^2^)] = 0.047	Hydrogen site location: inferred from neighbouring sites
*wR*(*F*^2^) = 0.140	H-atom parameters constrained
*S* = 1.03	*w* = 1/[σ^2^(*F*_o_^2^) + (0.0793*P*)^2^ + 0.4801*P*] where *P* = (*F*_o_^2^ + 2*F*_c_^2^)/3
2418 reflections	(Δ/σ)_max_ < 0.001
193 parameters	Δρ_max_ = 0.42 e Å^−^^3^
124 restraints	Δρ_min_ = −0.22 e Å^−^^3^

### Special details

**Table:**

Geometry. All e.s.d.'s (except the e.s.d. in the dihedral angle between two l.s. planes) are estimated using the full covariance matrix. The cell e.s.d.'s are taken into account individually in the estimation of e.s.d.'s in distances, angles and torsion angles; correlations between e.s.d.'s in cell parameters are only used when they are defined by crystal symmetry. An approximate (isotropic) treatment of cell e.s.d.'s is used for estimating e.s.d.'s involving l.s. planes.

### Fractional atomic coordinates and isotropic or equivalent isotropic displacement parameters (Å^2^)

**Table:**

	*x*	*y*	*z*	*U*_iso_*/*U*_eq_	Occ. (<1)
F1	0.5880 (4)	0.1389 (5)	0.7595 (4)	0.0560 (16)	0.722 (3)
F2	0.3734 (4)	0.1621 (3)	0.8509 (2)	0.0798 (12)	0.722 (3)
F3	0.4339 (3)	−0.0345 (2)	0.7799 (3)	0.0564 (7)	0.722 (3)
F4	0.3648 (2)	0.1294 (2)	0.66391 (16)	0.0572 (6)	0.722 (3)
F1'	0.5870 (8)	0.1500 (9)	0.7551 (6)	0.025 (2)	0.278 (3)
F2'	0.3434 (6)	0.1720 (6)	0.7957 (7)	0.065 (2)	0.278 (3)
F3'	0.4968 (6)	0.0503 (6)	0.9079 (4)	0.0664 (17)	0.278 (3)
F4'	0.4287 (12)	−0.0201 (7)	0.7334 (7)	0.099 (4)	0.278 (3)
N1	0.53432 (15)	0.55200 (13)	0.81766 (11)	0.0262 (3)	
N2	0.69734 (16)	0.64335 (14)	0.93417 (11)	0.0278 (3)	
C1	0.38756 (18)	0.46412 (16)	0.41995 (14)	0.0277 (4)	
H1	0.3101	0.4392	0.3655	0.033*	
C2	0.36751 (18)	0.44717 (16)	0.53553 (14)	0.0275 (4)	
H2	0.2761	0.4110	0.5595	0.033*	
C3	0.47960 (17)	0.48256 (14)	0.61695 (13)	0.0247 (3)	
C4	0.4550 (2)	0.45769 (16)	0.74175 (14)	0.0291 (4)	
H4A	0.3460	0.4621	0.7541	0.035*	
H4B	0.4900	0.3683	0.7619	0.035*	
C7	0.66006 (18)	0.53075 (16)	0.88270 (13)	0.0264 (4)	
C9	0.7447 (2)	0.40622 (18)	0.89444 (17)	0.0382 (4)	
H9A	0.6774	0.3329	0.8749	0.057*	
H9B	0.7850	0.3966	0.9735	0.057*	
H9C	0.8277	0.4069	0.8427	0.057*	
C8	0.8332 (2)	0.66710 (19)	1.00765 (15)	0.0368 (4)	
H8A	0.8437	0.5982	1.0657	0.055*	
H8B	0.8252	0.7522	1.0453	0.055*	
H8C	0.9214	0.6667	0.9611	0.055*	
C6	0.5949 (2)	0.73843 (17)	0.89965 (15)	0.0324 (4)	
H6	0.5962	0.8274	0.9229	0.039*	
C5	0.4932 (2)	0.68179 (17)	0.82714 (15)	0.0322 (4)	
H5	0.4089	0.7230	0.7894	0.039*	
B1	0.4413 (4)	0.0981 (4)	0.7654 (3)	0.0246 (8)	0.722 (3)
B1'	0.4639 (8)	0.0891 (8)	0.7968 (6)	0.026 (3)	0.278 (3)

### Atomic displacement parameters (Å^2^)

**Table:**

	*U*^11^	*U*^22^	*U*^33^	*U*^12^	*U*^13^	*U*^23^
F1	0.033 (3)	0.057 (2)	0.077 (3)	−0.0039 (17)	−0.0074 (19)	0.0112 (18)
F2	0.123 (3)	0.0547 (16)	0.0673 (16)	−0.0125 (16)	0.0595 (18)	−0.0215 (14)
F3	0.0532 (13)	0.0217 (9)	0.095 (2)	0.0044 (8)	0.0083 (13)	0.0100 (11)
F4	0.0433 (10)	0.0755 (13)	0.0509 (11)	−0.0056 (9)	−0.0156 (8)	0.0165 (9)
F1'	0.024 (5)	0.027 (3)	0.025 (3)	−0.003 (3)	0.005 (3)	−0.006 (2)
F2'	0.0158 (19)	0.035 (2)	0.146 (7)	0.0079 (17)	0.016 (3)	0.011 (4)
F3'	0.068 (3)	0.086 (4)	0.047 (3)	0.011 (3)	0.009 (2)	0.009 (2)
F4'	0.092 (5)	0.082 (6)	0.123 (7)	−0.031 (5)	0.011 (5)	−0.072 (5)
N1	0.0254 (7)	0.0273 (7)	0.0257 (7)	0.0001 (5)	−0.0009 (5)	0.0001 (5)
N2	0.0284 (7)	0.0327 (7)	0.0223 (6)	−0.0002 (5)	0.0007 (5)	−0.0026 (5)
C1	0.0228 (8)	0.0289 (8)	0.0305 (8)	−0.0023 (6)	−0.0060 (6)	−0.0037 (6)
C2	0.0214 (8)	0.0275 (8)	0.0334 (8)	−0.0034 (6)	−0.0007 (6)	−0.0019 (6)
C3	0.0241 (7)	0.0201 (7)	0.0294 (8)	0.0014 (6)	−0.0028 (6)	−0.0015 (6)
C4	0.0296 (8)	0.0268 (8)	0.0303 (8)	−0.0052 (6)	−0.0047 (6)	0.0003 (6)
C7	0.0279 (8)	0.0297 (8)	0.0215 (7)	0.0009 (6)	0.0011 (6)	0.0010 (6)
C9	0.0391 (10)	0.0313 (9)	0.0432 (10)	0.0078 (7)	−0.0079 (8)	0.0001 (7)
C8	0.0362 (9)	0.0441 (10)	0.0291 (8)	−0.0030 (8)	−0.0075 (7)	−0.0070 (7)
C6	0.0351 (9)	0.0283 (8)	0.0338 (9)	0.0043 (7)	0.0031 (7)	−0.0039 (7)
C5	0.0320 (9)	0.0290 (8)	0.0353 (9)	0.0061 (7)	−0.0013 (7)	0.0007 (7)
B1	0.0270 (16)	0.0229 (14)	0.0234 (18)	0.0008 (11)	−0.0033 (14)	−0.0002 (12)
B1'	0.022 (4)	0.032 (4)	0.023 (5)	0.005 (3)	−0.012 (3)	−0.006 (3)

### Geometric parameters (Å, °)

**Table:**

F1—B1	1.377 (4)	C2—C3	1.392 (2)
F2—B1	1.364 (4)	C2—H2	0.9500
F3—B1	1.368 (4)	C3—C1^i^	1.392 (2)
F4—B1	1.376 (4)	C3—C4	1.511 (2)
F1'—B1'	1.373 (7)	C4—H4A	0.9900
F2'—B1'	1.368 (6)	C4—H4B	0.9900
F3'—B1'	1.377 (6)	C7—C9	1.483 (2)
F4'—B1'	1.368 (7)	C9—H9A	0.9800
N1—C7	1.339 (2)	C9—H9B	0.9800
N1—C5	1.383 (2)	C9—H9C	0.9800
N1—C4	1.467 (2)	C8—H8A	0.9800
N2—C7	1.333 (2)	C8—H8B	0.9800
N2—C6	1.379 (2)	C8—H8C	0.9800
N2—C8	1.468 (2)	C6—C5	1.340 (2)
C1—C2	1.386 (2)	C6—H6	0.9500
C1—C3^i^	1.392 (2)	C5—H5	0.9500
C1—H1	0.9500		
			
C7—N1—C5	109.11 (14)	C7—C9—H9C	109.5
C7—N1—C4	126.85 (14)	H9A—C9—H9C	109.5
C5—N1—C4	123.94 (14)	H9B—C9—H9C	109.5
C7—N2—C6	109.33 (14)	N2—C8—H8A	109.5
C7—N2—C8	125.87 (15)	N2—C8—H8B	109.5
C6—N2—C8	124.58 (15)	H8A—C8—H8B	109.5
C2—C1—C3^i^	120.51 (14)	N2—C8—H8C	109.5
C2—C1—H1	119.7	H8A—C8—H8C	109.5
C3^i^—C1—H1	119.7	H8B—C8—H8C	109.5
C1—C2—C3	120.79 (15)	C5—C6—N2	107.20 (15)
C1—C2—H2	119.6	C5—C6—H6	126.4
C3—C2—H2	119.6	N2—C6—H6	126.4
C2—C3—C1^i^	118.69 (15)	C6—C5—N1	107.02 (15)
C2—C3—C4	118.92 (14)	C6—C5—H5	126.5
C1^i^—C3—C4	122.36 (14)	N1—C5—H5	126.5
N1—C4—C3	112.68 (13)	F2—B1—F3	111.0 (3)
N1—C4—H4A	109.1	F2—B1—F4	107.7 (3)
C3—C4—H4A	109.1	F3—B1—F4	108.2 (3)
N1—C4—H4B	109.1	F2—B1—F1	110.5 (3)
C3—C4—H4B	109.1	F3—B1—F1	111.0 (3)
H4A—C4—H4B	107.8	F4—B1—F1	108.3 (3)
N2—C7—N1	107.34 (14)	F2'—B1'—F4'	110.1 (6)
N2—C7—C9	125.89 (15)	F2'—B1'—F1'	110.9 (6)
N1—C7—C9	126.76 (15)	F4'—B1'—F1'	110.0 (6)
C7—C9—H9A	109.5	F2'—B1'—F3'	108.4 (6)
C7—C9—H9B	109.5	F4'—B1'—F3'	108.0 (6)
H9A—C9—H9B	109.5	F1'—B1'—F3'	109.5 (6)
			
C3^i^—C1—C2—C3	−0.2 (3)	C8—N2—C7—C9	3.3 (3)
C1—C2—C3—C1^i^	0.2 (3)	C5—N1—C7—N2	1.03 (18)
C1—C2—C3—C4	−177.78 (15)	C4—N1—C7—N2	177.39 (14)
C7—N1—C4—C3	−104.65 (18)	C5—N1—C7—C9	−177.97 (17)
C5—N1—C4—C3	71.2 (2)	C4—N1—C7—C9	−1.6 (3)
C2—C3—C4—N1	−150.75 (15)	C7—N2—C6—C5	0.69 (19)
C1^i^—C3—C4—N1	31.3 (2)	C8—N2—C6—C5	175.47 (16)
C6—N2—C7—N1	−1.06 (18)	N2—C6—C5—N1	−0.05 (19)
C8—N2—C7—N1	−175.76 (15)	C7—N1—C5—C6	−0.61 (19)
C6—N2—C7—C9	177.95 (17)	C4—N1—C5—C6	−177.10 (15)

Symmetry codes: (i) −*x*+1, −*y*+1, −*z*+1.
